# A rare case report of bilateral recurrent inguinal hernia due to persistent Müllerian duct syndrome treated by transabdominal preperitoneal repair

**DOI:** 10.1097/MD.0000000000019079

**Published:** 2020-02-14

**Authors:** Kan Tanabe, Shinichiro Mori, Yoshiaki Kita, Masumi Wada, Baba Kenji, Omoto Itaru, Arigami Takaaki, Iino Satoshi, Maemura Kosei, Shoji Natsugoe

**Affiliations:** a Department of Digestive Surgery, Breast and Thyroid Surgery Graduate School of Metical Sciences Kagoshima University, Kagoshima, Japan; b Department of Digestive Surgery, Imamura General Hospital.

**Keywords:** inguinal hernia, laparoscopic, persistent Müllerian duct syndrome, PMDS, TAPP, transabdominal preperitoneal repair

## Abstract

**Introduction:**

Persistent Müllerian duct syndrome (PMDS) is a rare disease occurring in men with an otherwise completely normal phenotype, in which female internal sex organs are present, including a uterus, fallopian tubes, cervix, and vagina. We report a case of bilateral recurrent inguinal hernia due to PMDS treated by transabdominal preperitoneal repair (TAPP).

**Patient concerns:**

A 72-year-old male presented with a complaint of swelling on both sides of the groin. The patient had undergone bilateral inguinal hernia suture repair 50 years ago.

**Diagnosis:**

Bilateral recurrent inguinal hernia

**Interventions:**

TAPP was performed. There was a fibrous structure linking the left and right hernia orifice and a muscular structure in the hernia sac on the left. We noticed that the muscular structure was a vagina and fibrous structure was the salpinx, and we diagnosed the patient with PMDS. Supravaginal hysterectomy and right salpingectomy were performed. After that a preperitoneal mesh repair was performed for bilateral inguinal hernia.

**Outcomes:**

Histologically, the diagnosis was confirmed as PMDS. The patient had an uneventful recovery.

**Conclusion:**

This case is the first case of bilateral recurrent inguinal hernia due to PMDS managed by TAPP.

## 1 Introduction

Persistent Müllerian duct syndrome (PMDS) is a rare disease occurring in men with an otherwise completely normal phenotype, in which female internal sex organs are present, including a uterus, fallopian tubes, cervix, and vagina.^[1,2]^ Approximately 200 cases of PMDS have reported, however hernia caused by PMDS was very rare.^[3]^ Preoperative diagnose of PMDS is difficult. We report a case bilateral recurrent inguinal hernia due to PMDS treated by transabdominal preperitoneal repair (TAPP).

## 2 Case report

**Figure 1:**
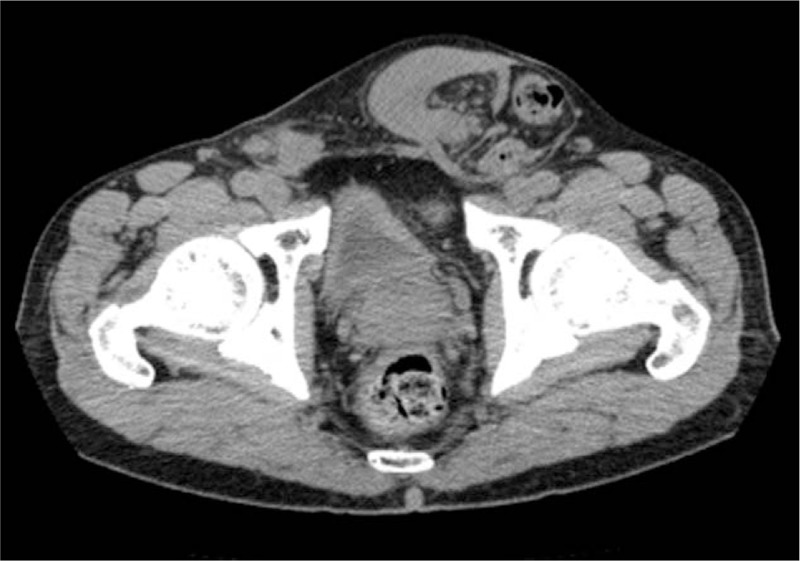
Computerized tomography. There were intestinal tract and solid tissue in hernia sac.

A 72-year-old male presented with a complaint of swelling on both sides of the groin for the past 40 years and left groin pain for 1 month. The pain reduced when the patient was in the supine position. The patient had undergone bilateral inguinal hernia suture repair 50 years ago. He had no sexual dysfunction and was fertile; he was married with three sons. On physical examination, a swelling of approximately 10 cm in diameter was found in the left groin extending into the left scrotum and a swelling of approximately 3 cm in diameter was found in the right groin. The swelling was reducible on both sides. Laboratory data were normal. Computed tomography showed a herniation in the left groin, but not in the right. The hernia contents seemed to be the intestinal tract and solid tissue considered the omentum (Fig. 1).

After confirming the patient's fitness to undergo anesthesia, surgery was planned. TAPP was performed. There was a fibrous structure linking the left and right hernia orifice and a muscular structure in the hernia sac on the left (Fig. 2 A). It was difficult to separate the peritoneal cavity and the structure of the fiber and muscle. The muscular structure was continuous in the pelvic cavity and adherent (Fig. 2 B). We consulted a pediatric urologist and noticed that the muscular structure was a vagina and fibrous structure was a salpinx, and we diagnosed the patient with PMDS (Fig. 2 C). We explained it to patient family and it was decided to resect a vagina and a salpinx for hernia repair. After dissection of the uterus, right salpinx, and the upper part of the vagina, supravaginal hysterectomy and right salpingectomy were performed. We divided the vagina with a linear stapler after dissecting the vagina as much as possible. Following which a preperitoneal mesh repair was performed for bilateral inguinal hernia. The mass comprised of Müllerian duct derivatives with a rudimentary uterus and right salpinx (Fig. 2 D). The operative time was 545 min, and the amount of bleeding was 50 mL. The patient had an uneventful recovery, with 7 postoperative days. Histologically, the tissue was accompanied by a salpinx-like structure, thick muscle tissues, and endometrial membranes, and the diagnosis was confirmed as PMDS (Fig. 3). There was no postoperative recurrence for 1 year and 10 months.

**Figure 2:**
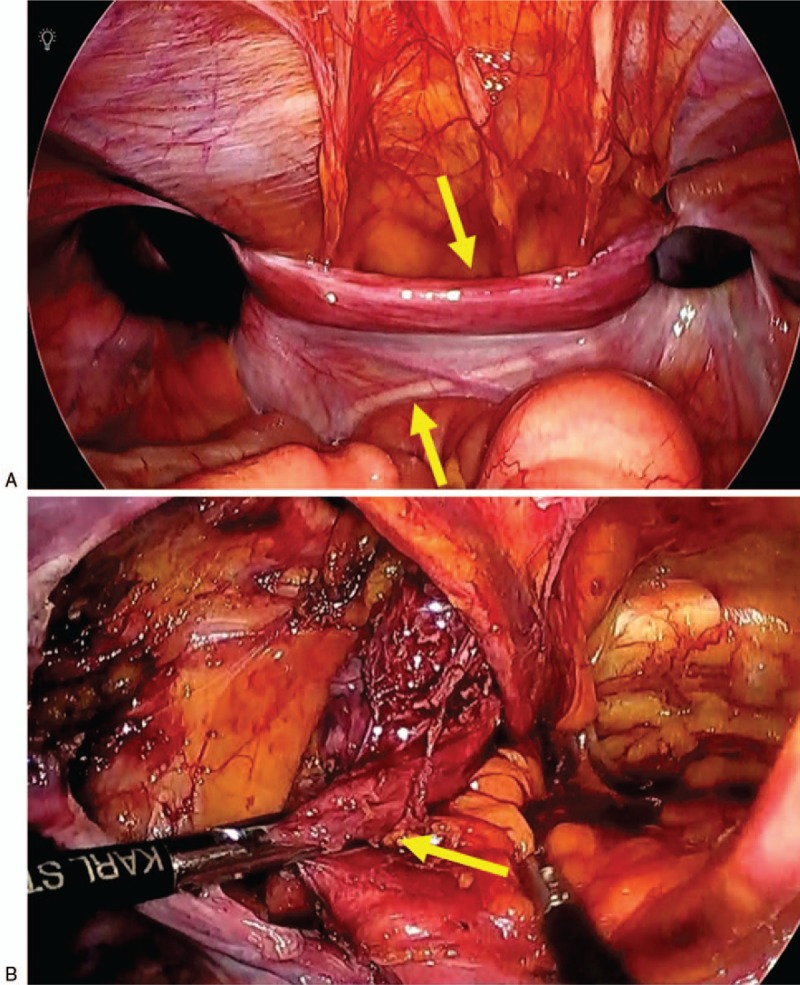
Intraoperative findings and specimen. (A) A fibrous structure linked the left and right hernia orifices and the right spermatic duct was crossing the median. (B) A muscular structure was found in the hernia sac on the left. (C) Schema of the intraoperative findings. (D) Extracted specimen.

**Figure 2 (Continued):**
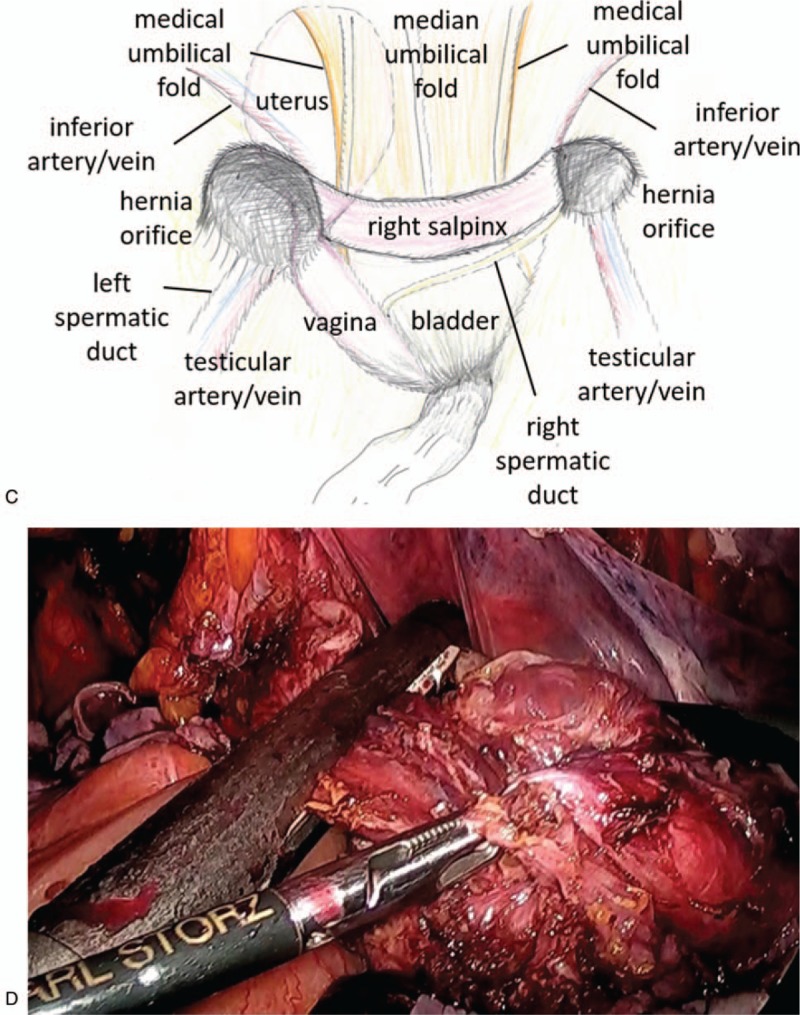
Intraoperative findings and specimen. (A) A fibrous structure linked the left and right hernia orifices and the right spermatic duct was crossing the median. (B) A muscular structure was found in the hernia sac on the left. (C) Schema of the intraoperative findings. (D) Extracted specimen.

**Figure 3:**
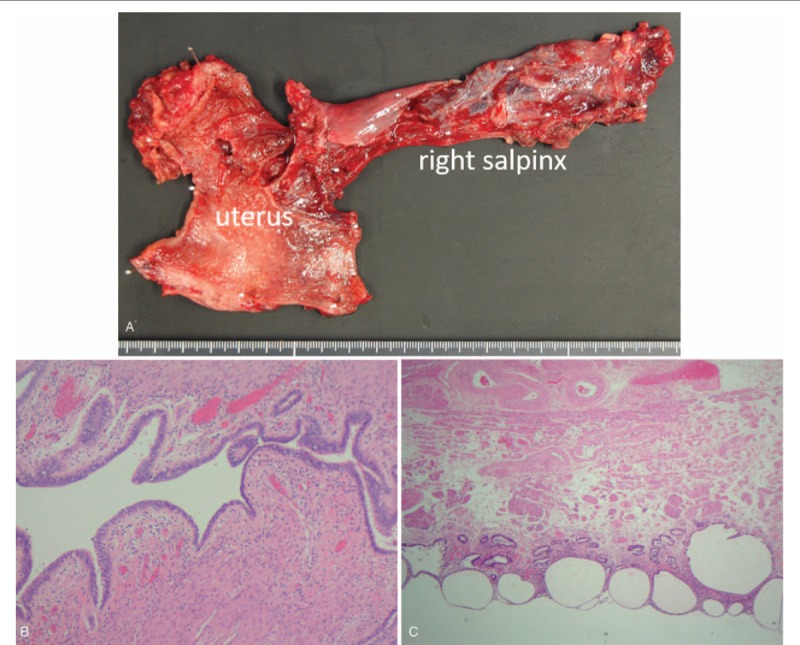
Extracted specimen and pathological findings. (A) Extracted specimen. (B) A salpinx-like structure was covered ciliated columnar epithelium. (C) The thick muscle tissues and endometrial membranes.

## 3 Discussion

PMDS is defined as the presence of Müllerian duct derivatives, uterus, and salpinxes in an otherwise normal masculinized 46, XY patient.^[1]^ The external virilization is complete but due to anti-Müllerian hormone deficiency or anti-Müllerian hormone receptor disorder, the Müllerian ducts do not regress and coexist with the testes and male excretory ducts.^[2]^ The removal of Müllerian organs is controversial because of two complications of PMDS: infertility and malignancy. Infertility is the most frequent complication of PMDS; however, our patient was married and already had three sons. Malignant degeneration of PMDS is very rare but there are a few existing reports of such cases.^[2,4]^ PMDS is usually discovered during surgery for cryptorchidism or inguinal hernia.^[5–7]^ Therefore, there are most reports in infants and children.^[8,9]^ However, this case was found to be an adult. Specifically, there are several reports of PMDS being discovered during open inguinal hernia repair,^[5,7,8]^ but there is no report of TAPP for such cases to date. To the best of our knowledge, this case is the first case of bilateral recurrent inguinal hernia due to PMDS treated by TAPP. PMDS diagnostics can be missed in open technique, and diagnostic laparoscopy is useful.^[10]^ Fifty years ago, the present patient had undergone bilateral inguinal hernia repair and had been bothered by swelling in the bilateral groin for the past 40 years. Thus, there was a possibility of recurrence if the contents in the hernia sac were not removed. PMDS considered to be the pathogenesis of recurrence of bilateral inguinal hernia. Moreover, the completion of TAPP would have been impossible unless the contents of the inguinal hernia were removed. Therefore, we decided to remove the Müllerian organs to complete preperitoneal repair. The use of the laparoscopic approach for this case was very effective because we could confirm bilateral recurrent hernia and diagnose PMDS, leading to a complete bilateral hernia repair with a minimally invasive surgical technique.

In conclusion, patients presenting with bilateral recurrent inguinal hernia with contents to cross both sides exist should be properly evaluated. In such cases, PMDS should be considered as a differential diagnosis. Moreover, laparoscopic surgery should be considered by surgeons for the diagnosis and treatment of patients with PMDS.

## Acknowledgments

The authors would like to acknowledge the patient for allowing this case to be published. The authors would like to acknowledge Editage (www.editage.com) for English language editing.

## Author contributions

**Conceptualization:** Kan Tanabe, Shinichiro Mori.

**Resources:** Yoshiaki Kita, Masumi Wada, Baba Kenji.

**Supervision:** Omoto Itaru, Arigami Takaaki, Iino Satoshi, Maemura Kosei, Shoji Natsugoe.

**Writing – original draft:** Kan Tanabe.

**Writing – review & editing:** Shinichiro Mori, Shoji Natsugoe.
